# Modified Coronally Advanced Flaps: A Systematic Review and Meta-Analysis

**DOI:** 10.3390/dj13100477

**Published:** 2025-10-17

**Authors:** Miriana Gualtieri, Annarita Signoriello, Alessia Pardo, Diana Andreea Muresan, Alessandro Zangani, Paolo Faccioni, Giovanni Corrocher, Giorgio Lombardo

**Affiliations:** Dentistry and Maxillo-Facial Surgery Unit, Department of Surgery, Dentistry, Paediatrics and Gynaecology (DIPSCOMI), University of Verona, Piazzale L.A. Scuro 10, 37134 Verona, Italy; miriana.gualtieri@studenti.univr.it (M.G.); dianaandreea.muresan@studenti.univr.it (D.A.M.); alessandro.zangani@univr.it (A.Z.); paolo.faccioni@univr.it (P.F.); giovanni.corrocher@univr.it (G.C.); giorgio.lombardo@univr.it (G.L.)

**Keywords:** gingival recessions, mucogingival surgery, connective tissue grafts, xenogeneic grafts

## Abstract

**Background**: Gingival recession (GR) is defined as the exposure of the root surface due to the gingival margin shifting apically from the cemento-enamel junction. Current effective management of defects related to GR relies on root coverage periodontal plastic surgery (RCPPS), using the Modified Coronally Advanced Flap (mCAF) with an envelope design. Recent literature also reported the association of different biomaterials to the mCAF procedure. In light of these considerations, a systematic review (SR) was conducted to determine and compare the efficacy of all mCAF adjunctive techniques for the treatment of multiple adjacent GR-type (MAGR) defects. **Methods**: An electronic search was conducted in 2025 on studies published between 2013 and 2025, using PubMed, Scopus, Web of Science, and Cinahl Complete, to address the focused question: “which is the efficacy of different mCAF adjunctive techniques for the treatment of multiple adjacent GR-type defects, in terms of root coverage (RC), esthetic outcomes, and keratinized tissue (KT) augmentation?”. Randomized controlled trials with a minimum follow-up of 6 months with ≥ 5 patients treated for coverage of MAGR were included. Risk of bias was assessed with RoB 2 Tool. A meta-analysis was performed using RevMan5.4 software and the level of evidence of included studies was analyzed with GRADEPro GDT. **Results**: A total of 17 studies were included in the SR, 9 of which evaluating mCAF + sCTG (subepithelial connective tissue graft) vs. mCAF adjunctive techniques [Collagen Matrix (CM), xenogeneic acellular dermal matrix (XADM), Platelet-Rich Fibrin (PRF), Enamel Matrix Derivatives (EMD), sCTG harvested double blade scalpel] were then included in the meta-analysis. The primary outcomes of complete root coverage (CRC) and keratinized tissue width variation (ΔKTW) were statistically significant ([CRC: Odds Ratio (OR) 1.70; 95% CI (confidence interval) 1.18, 2.44; *p* = 0.004]; [ΔKTW: SMD (standardized mean difference) 0.37; 95% CI 0.11, 0.63; *p* = 0.005]) in favor of mCAF + CTG. Meanwhile, no statistically significant difference was observed in terms of RES. The certainty assessment highlighted relevant results: despite the lack of evidence in the long-term, a high level of evidence showed that sCTG was more effective than XADM in terms of CRC (*p* = 0.002) and ΔKTW (*p* = 0.0001). A low level of evidence revealed that sCTG achieved a greater ΔKTW compared to CM (*p* = 0.0006). Although no significant differences were observed, a low level of evidence suggested that mCAF + EMD and mCAF + sCTG (DBS) may provide good results. To date, only one RCT showed long-term stable results of CTG in terms of RC. **Conclusions**: The association of sCTG to mCAF demonstrated better results in terms of RC and KTW augmentation in short- and medium-term follow-ups. Long-term studies are needed to confirm the efficacy of the other mCAF adjunctive techniques, considering limitations due to heterogeneity in follow-ups, distribution of techniques analyzed, and different study designs. Registration in PROSPERO (International prospective register of systematic reviews) was performed with ID CRD420251085823.

## 1. Introduction

Gingival recession (GR), defined as the exposure of the root surface due to the apical shift in the gingival margin from the cemento-enamel junction [1,2], is usually associated with impaired esthetics. If left untreated, it can lead to dental hypersensitivity, poor plaque control, root caries, and even loss of periodontal support [2,3]. These functional and esthetic issues are more frequently perceived in younger individuals, especially in the case of deeper GR and involved anterior areas, even in the presence of non-carious cervical lesions [4].

The development of GR is related to various etiological factors, mainly inflammatory, anatomical, or mechanical, such as plaque-induced periodontal disease [5,6,7], viral infections [8], traumatic tooth brushing [9], aberrant frenum attachment [10], thin gingival phenotype [11], malocclusion and dental crowding [12], subgingival restorative margin invading the biological width [13,14], orthodontic treatment [15,16], and lip/tongue piercings [17].

Multiple adjacent recessions are even more challenging defects, as the surgical field is larger and more anatomical variations may be present (prominent roots, shallow vestibules, defect size) [18]. Current effective management of these defects relies on root coverage periodontal plastic surgery (RCPPS). Several randomized clinical trials (RCTs) and systematic reviews (SRs) analyzed the efficacy of the most used RCPPS techniques, e.g., the Coronally Advanced Flap (CAF) with or without subepithelial connective tissue graft (sCTG), and the Coronally Advanced Tunnel technique (CAT) with or without sCTG [19,20,21,22,23]. Successful treatment of GR aims to eliminate etiologic factors related to the onset and progression of the defect, characterizing the phenotype of the recession site and adjacent areas, and selecting the most suitable option [24]. In this context, the CAF is commonly considered the gold standard to treat GR [19,25], as it typically renders the best cost–benefit treatment outcomes in terms of complete root coverage (CRC), mean root coverage (MRC), keratinized tissue width (KTW) gain, and long-term stability of treatment outcomes.

Nevertheless, vertical releasing incisions performed with the CAF may disrupt its vascularity or negatively influence the esthetic outcome due to keloid/scar formation [26]. To address these drawbacks, a Modified Coronally Advanced Flap (mCAF) was proposed, with the employment of an envelope design, characterized by a sub-marginal oblique incision in the papillary area, anticipating the rotational movement of the surgical papilla during coronal advancement [27], without the need for vertical incisions. Further studies reported improved clinical and esthetic outcomes with the additional use of connective tissue graft (CTG) to this novel approach, compared to the conventional bilaminar technique [28,29].

Recent literature focused on the association of different biomaterials as an adjunct to the flap alone: acellular dermal matrix (ADM) [30], xenogenic acellular dermal matrix (mCAF + XADM) [31,32,33,34], Platelet-Rich Fibrin (mCAF + PRF) [35], Collagen Matrix (mCAF + CM) [36,37], barrier membrane (mCAF + PRF membrane, mCAF + placental membrane) [38,39], hyaluronic acid (HA) [40,41,42], other adjunctive techniques, e.g., the orthodontic button application (mCAF + OB) [43,44], or the use of a microsurgical approach [45].

Based on the hypothesis that these mCAF adjunctive techniques may provide efficacy not only in the short-, but also in the long-term treatment of multiple adjacent GR-type defects, a SR was conducted in 2025 to determine and compare their outcomes in terms of root coverage (RC), esthetic outcomes, and keratinized tissue (KT) augmentation. The novelty of this SR lies in its specific clinical focus on these latest mCAF adjunctive techniques, with the aim of enriching comparison between them and mCAF alone.

## 2. Materials and Methods

This study was conducted in 2025 in accordance with the PRISMA guidelines and checklist (2020) [46]. The review was conducted according to the population, intervention, comparison, outcome, and study design (PICOS) format [47]. Registration in PROSPERO (International prospective register of systematic reviews) was performed with ID CRD420251085823 on 4 July 2025.

### 2.1. Search Strategy

An electronic search was conducted using the following databases: PubMed, Scopus, Web of Science, and Cinahl Complete, considering studies published between 2013 and 2025.

In MEDLINE (PubMed) the following search terms were used:

(1) ((multiple recessions[Title/Abstract]) OR (multiple recession-type defects[Title/Abstract])) AND (treatment[Title/Abstract]);
(2) Modified Coronally Advanced Flap[Title/Abstract].

In Scopus the following search terms were used:

(1) ((TITLE-ABS-KEY (multiple AND recessions) OR TITLE-ABS-KEY (multiple AND recession-type AND defects)) AND TITLE-ABS-KEY (treatment));
(2) TITLE-ABS-KEY(modified AND coronally AND advanced AND flap).

In Web of Science the following search terms were used:

(1) ((TI = (multiple recessions)) OR TI = (multiple recession-type defects)) AND TI = (treatment);
(2) TI = (Modified Coronally Advanced Flap).

In Cinahl Complete the following search terms were used:

(1) TI multiple recessions OR TI multiple recession-type defects AND TI treatment;
(2) TI Modified Coronally Advanced Flap.

#### Focused Question

The focused question was defined as follows:

“What is the efficacy of different mCAF adjunctive techniques for the treatment of multiple adjacent GR-type defects, in terms of root coverage (RC), esthetic outcomes, and keratinized tissue (KT) augmentation?”. Furthermore, the aim of the literature search was to evaluate the long-term results of these mCAF adjunctive techniques.

### 2.2. Inclusion Criteria and Selection Process

Study selection criteria based on the PICOS (Population, Intervention, Comparison, Outcomes, and Study design) framework were as follows.

#### 2.2.1. Types of Participants

Based on the defined focused question, studies with patients with at least two gingival recession (GR) defects were included. Miller classification [48] and recession-type (RT) classification based on the interdental clinical attachment level [49] were considered.

#### 2.2.2. Types of Interventions

The following root coverage surgical procedures for the treatment of multiple gingival recessions were considered:

- mCAF (Modified Coronally Advanced Flap): An envelope type of flap proposed by Zucchelli and De Sanctis [27], characterized by a horizontal incision consisting of oblique submarginal incisions in the interdental areas, which continued with the intra-sulcular incision at the recession defects. This kind of design anticipates the rotation of the surgical papilla;
- mCAF + sCTG (subepithelial connective tissue graft): The envelope type of CAF proposed by Zucchelli and De Sanctis [27], with site-specific subepithelial connective tissue graft [31,32,33,34,35,45,50];
- mCAF + PRF (Platelet-Rich Fibrin): PRF is a second-generation platelet concentrate prepared from centrifuged blood in glass or titanium tube (T-PRF). It is associated with the mCAF technique in the form of PRF membrane [35,38,50];
- mCAF + CM (Collagen Matrix): CM is a resorbable, three-dimensional (3D) matrix of pure type I and III porcine collagen obtained with standardized and controlled manufacturing processes without cross-linking or chemical treatment [36,51];
- mCAF + XADM (xenogeneic acellular dermal matrix): XADM is 3D porcine-derived acellular dermal matrix of collagen and elastin, which undergoes several purification processes that remove the full rejection potential of the tissue [31,32,33,34];
- mCAF + OB (Orthodontic Button) application: After the coronally displacement, sutures were stabilized with orthodontic buttons [43,44];
- mCAF + placental membrane: A placental allograft deriving from amnion and chorion was used for the first time to compare mCAF + placental membrane to mCAF alone in the treatment of gingival recession [39];
- mCAF + EMD (Enamel Matrix Derivatives): EMD was evaluated for its potential both in regeneration of intrabony defects and in root coverage surgical procedures [52].

#### 2.2.3. Types of Studies

Randomized controlled trials with a minimum follow-up of 6 months with ≥5 patients were included. Retrospective studies, prospective studies, in vitro studies, animal studies, case reports, case series, narrative reviews, and systematic reviews were excluded.

#### 2.2.4. Types of Measures: Primary and Secondary Outcomes

The variables sought in each study were defined as follows.

Primary outcomes were as follows:

- Complete root coverage (CRC) [53,54]: A percentage value describing the number of sites, with respect to the total number of sites treated, that obtained a complete radicular covering at a given time of follow-up. The formula is reported as CRC = (n. of sites with CRC)/(total n. of sites treated) × 100%;
- Root esthetic score (RES) [55,56,57]: A score evaluating level of the gingival margin, marginal tissue contour, soft tissue texture, mucogingival junction alignment, and gingival color. Regarding assessment of the final position of the gingival margin, 3 points are given for partial root coverage and 6 points for complete root coverage, 0 points are assigned when the final position of the gingival margin is equal or apical to the previous recession. One point is assigned for each of the other four variables. Thus, 10 points is the perfect score;
- Differential keratinized tissue width (ΔKTW) [58,59], where KTW is the distance from the free gingival margin to the mucogingival junction.

Secondary outcomes were as follows:

- Mean root coverage (MRC) [60]: A percentage value that describes the rate of reduction in the recession compared to the initial recession;
- Recession depth reduction (RDR) [61,62]: A value in mm which describes the difference between the recession depth measure at a given follow-up and the measure of the initial recession depth;
- Differential gingival thickness (ΔGT) [31,34,59], where GT is a measurement in mm which indicates the thickness of the attached gingiva;
- Duration of surgery, measured in minutes.

### 2.3. Data Collection, Extraction, and Management

Titles of the studies assessed through the search previously described (using the predetermined inclusion and exclusion criteria) were screened by two independent examiners (M.G. and A.S.), to minimize the risk of reviewer bias. Duplicates were deleted. Data collection from the included reports was performed by the two reviewers, who independently worked with an Excel spreadsheet. In the case of disagreement regarding eligibility, the two reviewers analyzed the title jointly to reach a final decision concerning inclusion or exclusion. Articles identified as potentially useful through analysis of the title were then selected for a more in-depth investigation by reading the abstract. In the examination of the abstract, attention was paid to assess the compliance of the study with the inclusion criteria. The selected studies were then saved as a digital version and submitted for full-text reading. In this way, only articles that fulfilled all criteria of the selection process were finally included for data extraction.

Data extraction and management were performed by filling in a table in the Excel spreadsheet with the following data: Titles, Authors, Year, Design, N° Patients, N° Sites, Surgery Test vs. Control, N° Patients in each group, N° Sites in each group, CRC (%), % RC, RDR, ΔKTw, ΔGT, Duration of the surgery (min), RES, Follow-up.

#### 2.3.1. Assessment of Risk of Bias of the Included Studies

According to the Risk of Bias 2 (RoB 2) Tool [63], the following parameters were adopted for the evaluation of risk of bias for RCTs: random sequence generation and allocation concealment (selection bias), blinding of participants and personnel (performance bias), blinding of outcome assessment (detection bias), incomplete outcome data (attrition bias), selective reporting (reporting bias). In the event of a disagreement between the same two reviewers (M.G. and A.S.), additional discussion was used to reach a consensus. All data were represented through a risk of bias graph and a risk of bias summary.

All data were graphically represented through a traffic-light plot and a summary plot by uploading the data into the Robvis web application (visualization tool) [64].

#### 2.3.2. Quantitative Data Synthesis Method

A Meta-Analysis was carried out using RevMan5 software [65].

Primary and secondary outcomes were considered in performing the meta-analysis, examining both dichotomous variables (CRC) and continuous variables (MRC, GT gain, RDR, KTW gain, RES, duration of surgery).

For the dichotomous CRC data, the inverse variance method was applied with the risk ratio (RR) as the main effect measure. The random effects model was adopted, which considers variation between studies in addition to random error, allowing for generalization of results to a broader population. Results were presented with a 95% confidence interval, providing a precise estimate of the effect with a suitable level of certainty.

For continuous data (MRC, GT gain, RDR, KTW gain, and duration of surgery) the inverse variance method was also employed. However, in this case, the effect measure used was the standard mean difference (SMD). The magnitude of the SMD was interpreted as mild if SMD = 0.2, mean if SMD = 0.5 and high if SMD = 0.8.

The heterogeneity between studies was assessed with the heterogeneity index of Higgins (I^2^). Heterogeneity is considered low if I^2^ is less than 40%, moderate if I^2^ is between 40% and 70%, substantial if I^2^ is between 70% and 90%, and considerable if I^2^ is more than 90%. When the heterogeneity was low (I^2^ < 40%), a fixed effects model was applied; otherwise, a random effects model analysis was undertaken to account for variation between studies and obtain more robust estimates. Parallel-group and split-mouth studies were combined in the meta-analyses of treatment effects. The significance level was set at alpha = 0.05. Results were reported with a 95% confidence interval (CI) to indicate the precision of the estimates. All data were graphically represented using a forest plot.

Subgroups analysis was conducted considering the threshold of *p*-value as less than 0.1 [66]. Results were reported with the significance of subgroup differences at every level of heterogeneity: in case of moderate to high heterogeneity, meta-analysis was included, but the uncertainty in the evidence was acknowledged as inconsistency between individual trial results [66], thus these results were retained but interpreted with caution.

#### 2.3.3. Certainty Assessment

The certainty assessment was conducted using GRADEpro GDT [67].

The same two reviewers (M.G. and A.S.) evaluated the certainty of evidence based on the following factors: risk of bias, inconsistency, indirectness, imprecision, publication bias, large effect, plausible confounding, and dose–response gradient. In the event of disagreement between the two reviewers, additional discussion was used to reach a consensus.

## 3. Results

### 3.1. Results of the Study Search

**Figure 1:**
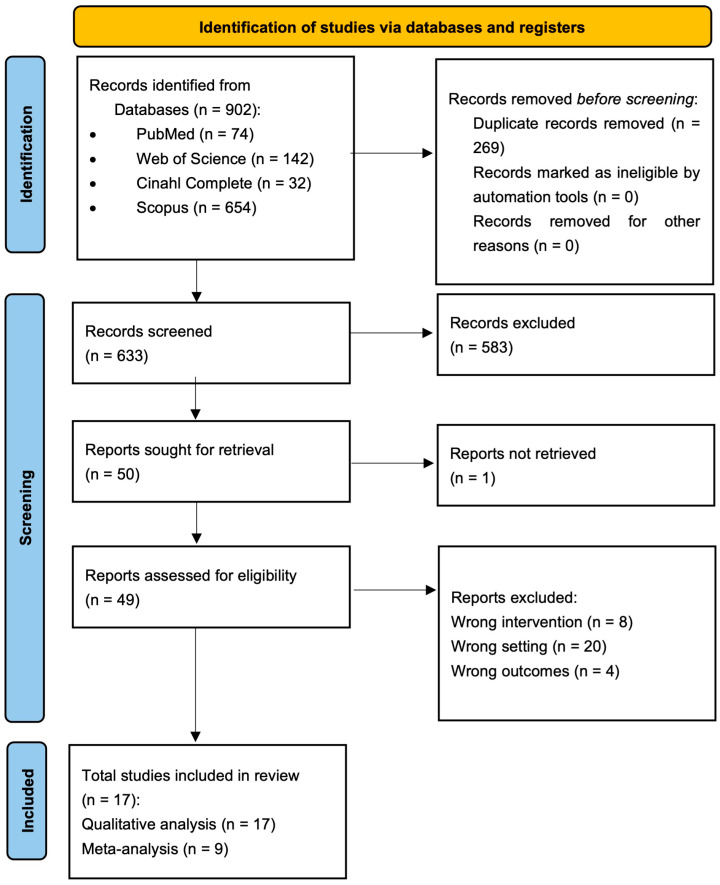
Flow chart summarizing the study selection procedure, constructed in accordance with PRISMA guidelines (2020).

The electronic search through the PubMed database identified 74 publications, the search using the Scopus database identified 654 publications, the Web of Science database identified 142 publications and the Cinahl Complete database identified 32 publications. Following the removal of all duplicates, 633 articles were identified up to the date of search (1 July 2025). Among these, after reading all titles and abstracts, 50 publications were sought for retrieval (one study was not retrieved). The full text analysis led to the exclusion of 32 articles with specified reasons, so the electronic search finally identified 17 articles, whose data are reported in Figure 1.

### 3.2. Characteristics of the Included Studies

All 17 studies included in the SR (see Table 1) were RCTs. Nine of these articles were performed with a split-mouth design, where each patient received both the test and control surgical interventions; in eight studies, a parallel-groups design was adopted, assigning patients to either the test or control surgery, respectively.

The number of patients included in each study ranged from 7 to 50 and the number of treated sites (number of GRs) ranged from 14 to 269.

The characteristics of the included studies are described in detail in Table 1.

### 3.3. Risk of Bias

**Figure 2:**
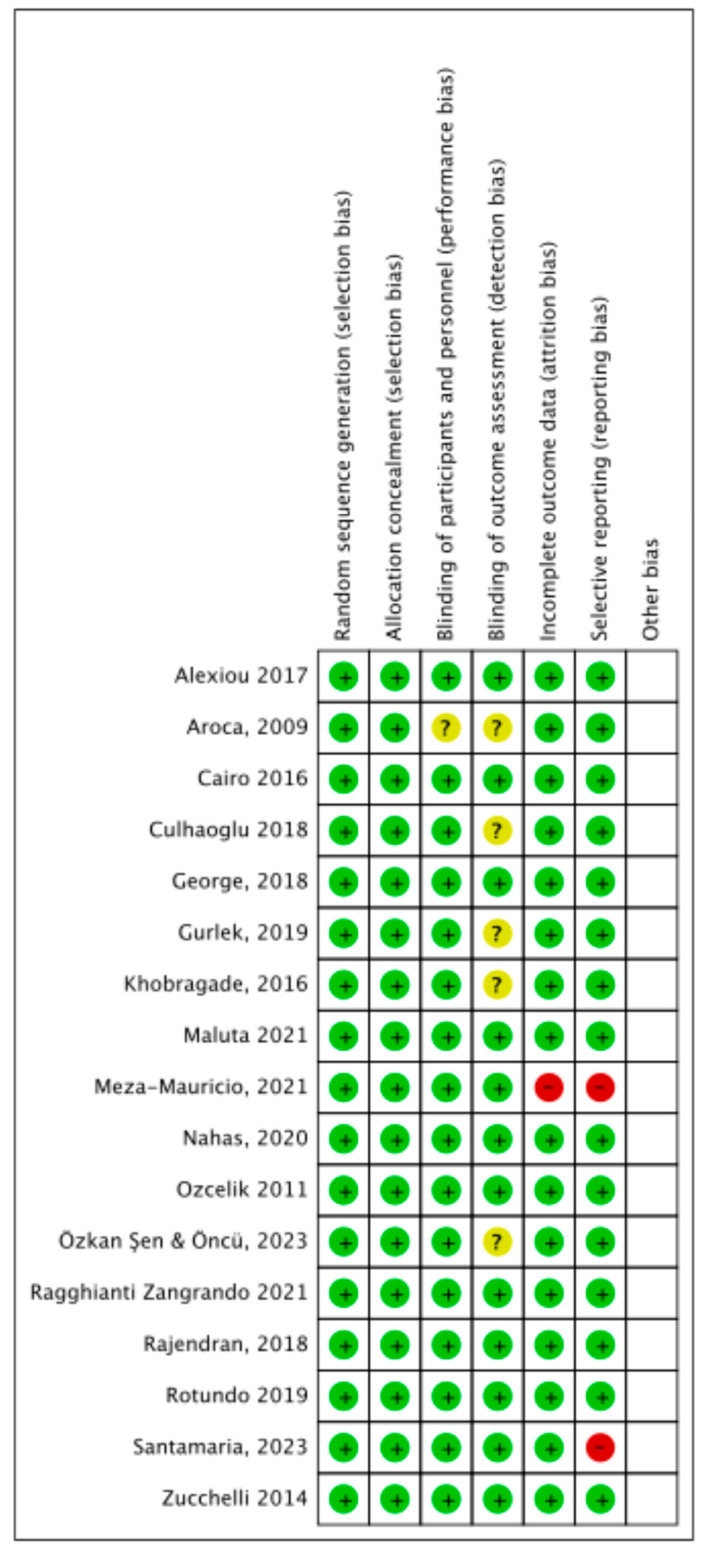
Risk of bias summary: Review authors’ judgements about each risk of bias item for each included study (RCTs); green color: low risk; yellow color: some concerns or unclear risk; red color: high risk ([31,32,33,36,38,39,43,44,50,52,68,69,70,71,72,73,74]).

**Figure 3:**
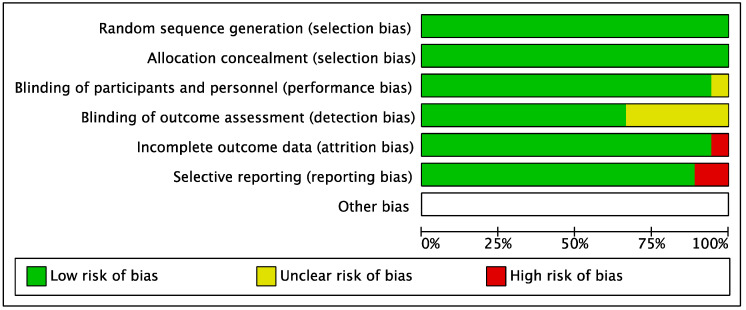
Risk of bias graph: Review authors’ judgements about each risk of bias item presented as percentages across all included studies.

Risk of bias (Figure 2 and Figure 3) in the included RCTs was evaluated using the data extracted from each trial.

All trials reported an adequate method of randomization and allocation concealment.

Sixteen trials reported adequate blinding of participants and personnel. One trial [38] was considered unclear as it did not mention the blinding of participants and personnel.

Regarding the blinding of outcomes assessment, 12 trials were considered at low risk of bias, while in 5 trials the blinding of the examiners was not mentioned, thus it was considered as unclear.

Most of the studies were at low risk of bias for incomplete outcomes data domain, except for one trial [32] in which esthetic (VAS) and pain (VAS) values were reported on a graphic scale, without reporting the mean values.

Selective reporting was considered at low risk of bias for 15 studies. The bias from selective outcomes reporting was considered high for 2 studies: respectively, in the first study [32], esthetic (VAS) and pain (VAS) values were reported on a graphic scale, while in the second study [31], the 3-month clinical measurements mentioned were not reported.

Results of individual studies included in the SR are described in Table 2. CRC was reported in 14 studies [31,32,33,35,36,38,44,52,68,70,71,72,73,74]. MRC was reported in 13 studies [31,32,35,36,38,44,50,52,69,70,71,72,73]. RDR was reported in 10 studies [31,32,33,36,39,68,70,71,72,73]. RES was reported by five studies [32,44,68,71,72]. ∆GT was reported in five studies [31,32,68,72,73]. ∆KTW was reported in 10 studies [31,32,33,36,39,68,70,71,72,73]. Duration of surgery was reported in five studies [31,36,68,73,74].

### 3.4. Synthesis of Results of Meta-Analysis

**Figure 4:**
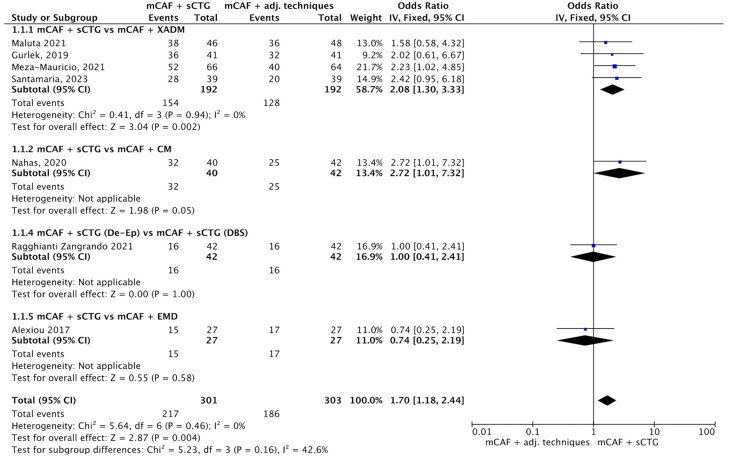
Forest plot of comparison: mCAF + sCTG vs. mCAF adjunctive techniques, outcome: CRC (%) ([31,32,33,36,52,70,72]).

**Figure 5:**
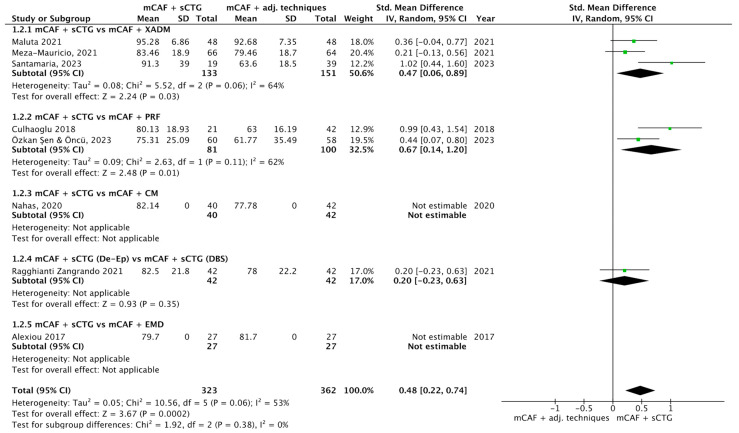
Forest plot of comparison: mCAF + sCTG vs. mCAF adjunctive techniques, outcome: mean root coverage (MRC) (%) ([31,32,35,36,50,69,70,72,73]).

**Figure 6:**
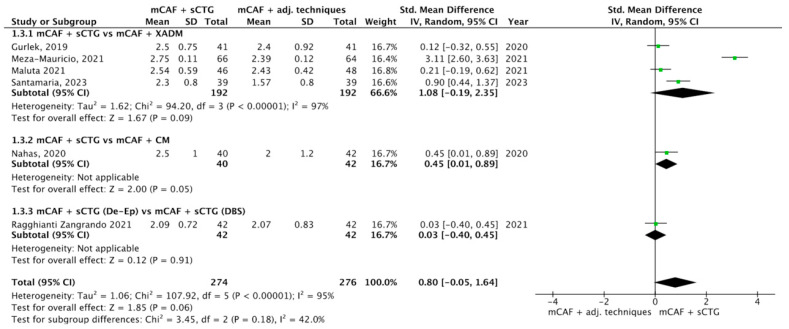
Forest plot of comparison: mCAF + sCTG vs. mCAF adjunctive techniques, outcome: recession depth reduction (RDR) (mm) ([31,32,33,36,70,72]).

**Figure 7:**
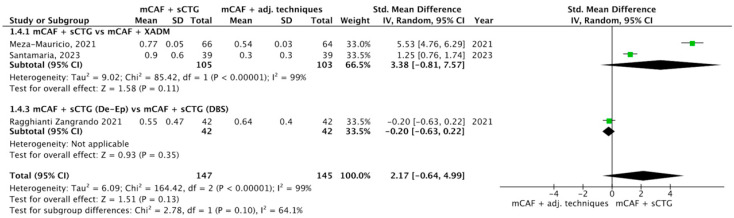
Forest plot of comparison: mCAF + sCTG vs. mCAF adjunctive techniques, outcome: gingival thickness (GT) gain (mm) ([31,32,72]).

**Figure 8:**
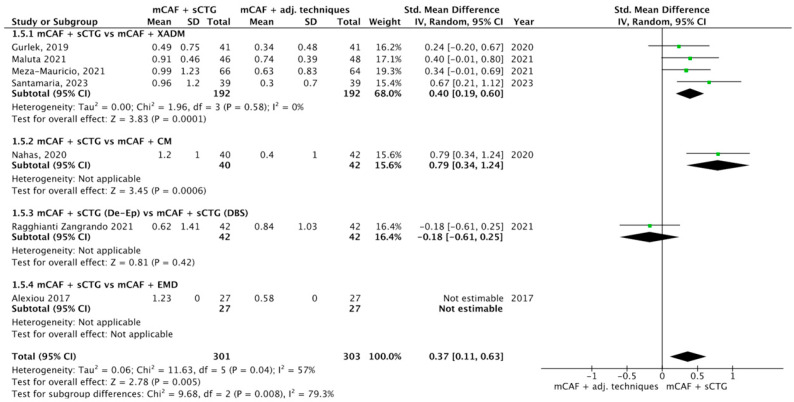
Forest plot of comparison: mCAF + sCTG vs. mCAF adjunctive techniques, outcome: keratinized tissue width (KTW) gain (mm) ([31,32,33,36,52,70,72]).

**Figure 9:**
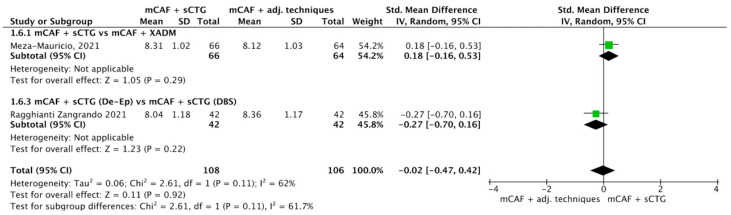
Forest plot of comparison: mCAF + sCTG vs. mCAF adjunctive techniques, outcome: root esthetic score (RES) ([32,72]).

**Figure 10:**
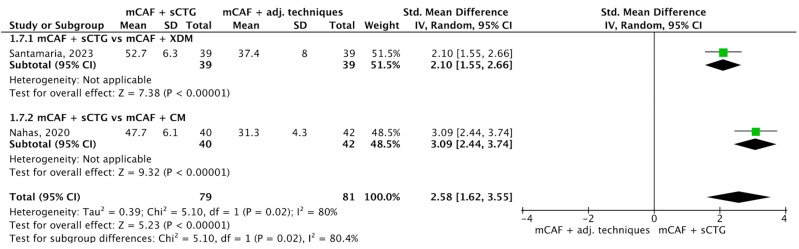
Forest plot of comparison: mCAF + sCTG vs. mCAF adjunctive techniques, outcome: duration of surgery ([31,36]).

Of the 17 included studies, 9 evaluating mCAF + sCTG vs. mCAF variation (CM, XADM, PRF, EMD, sCTG harvested double blade scalpel) were included in the following meta-analysis (Figures 4–10):

− mCAF + sCTG vs. mCAF + XADM [31,32,33,70];
− mCAF + sCTG vs. mCAF + CM [36];
− mCAF + sCTG vs. mCAF + PRF [35,50,69];
− mCAF + sCTG vs. mCAF + EMD [52];
− mCAF + sCTG (de-epithelialized) vs. mCAF + CTG (harvested double blade scalpel) [72].

The six parameters considered were CRC, MRC, RDR, KTW gain, GT gain, RES, duration of surgery. For each parameter, we created a forest plot to explore the efficacy of mCAF adjunctive techniques (test) compared to mCAF + sCTG (control).

When outcomes were reported, comparisons were divided into subgroups:

- mCAF + sCTG (control) vs. mCAF + XADM (test);
- mCAF + sCTG (control) vs. mCAF + CM (test);
- mCAF + sCTG (control) vs. mCAF + PRF (test);
- mCAF + sCTG (control) vs. mCAF + EMD (test);
- mCAF + sCTG (de-epithelialized) (control) vs. mCAF + CTG (harvested double blade scalpel) (test).

#### 3.4.1. CRC

Eight trials reported this outcome (see Figure 4) [31,32,33,36,52,70,72]. The I^2^ value was 0%, indicating no observed heterogeneity among studies; therefore, a fixed-effect model was applied. The overall Odds Ratio (OR) was 1.70 [95% CI: 1.18 to 2.44], with a *p*-value of 0.004, indicating a statistically significant difference in favor of mCAF + sCTG.

In the subgroup mCAF + sCTG vs. mCAF + XADM, 192 sites were treated in each group. The I^2^ was 0%, so heterogeneity was considered low, and a fixed-effect model was used. The difference between groups was statistically significant (*p* = 0.002) in favor of mCAF + sCTG. In the subgroups mCAF + sCTG vs. mCAF + CM, mCAF + sCTG (de-epithelialized) vs. mCAF + sCTG (DBS), and mCAF + sCTG vs. mCAF + EMD, the differences between groups were not statistically significant, thus the test for heterogeneity was not applicable in these cases.

The test for subgroup differences showed no statistically significant interaction between the subgroups (Chi^2^ = 5.23, degrees of freedom (df) = 3, *p* = 0.16), although a moderate degree of heterogeneity was observed (I^2^ = 42.6%), suggesting that the treatment effect was relatively consistent across the different types of substitutes evaluated.

#### 3.4.2. MRC

Nine trials reported this outcome (Figure 5) [31,32,35,36,50,69,70,72,73]. I^2^ value was 58%, which indicates moderate heterogeneity: for this reason, the random effect model was used. The SMD was 0.48 (95% CI from 0.22 to 0.74) with a *p*-value of 0.0002, indicating a statistically significant difference in favor of mCAF + sCTG.

In the subgroup mCAF + sCTG (151 sites) vs. mCAF + XADM (133 sites), I^2^ value was 64%, so the heterogeneity was considered moderate. The difference between groups was statistically significant in favor of sCTG (*p* = 0.03). Even more evident was the analysis of the subgroup mCAF + sCTG vs. mCAF + PRF, which showed a *p*-value of 0.01. In the subgroups mCAF + sCTG vs. mCAF + CM, mCAF + sCTG (de-epithelialized) vs. mCAF + sCTG (DBS), and mCAF + sCTG vs. mCAF + EMD, no significant difference was found, thus test for heterogeneity was not applicable in these cases.

The test for subgroup differences yielded a Chi^2^ value of 1.92, with 2 df (*p* = 0.38), and I^2^ = 0%, indicating that no statistically significant difference in treatment effects across the subgroups was found. The low I^2^ value suggests no heterogeneity between subgroups, implying that the treatment effect is consistent across these groups.

#### 3.4.3. RDR

Five trials reported this outcome (Figure 6) [31,32,33,36,70,72]. I^2^ value was 95%, which indicates considerable heterogeneity: for this reason, the random effect model was applied. The SMD was 0.80, with 95% CI from −0.05 to 1.64 and *p* = 0.06 (not statistically significant).

In the subgroup mCAF + sCTG vs. mCAF + XADM, 192 sites were treated with each technique. The difference between groups was not statistically significant, even if a higher value of RDR was recorded with the sCTG. In the subgroups mCAF + sCTG vs. mCAF + CM and mCAF + sCTG (de-epithelialized) vs. mCAF + sCTG (DBS), the differences between groups were not statistically significant, thus test for heterogeneity was not applicable in these cases.

The test for subgroup differences yielded a Chi^2^ value of 3.45 with 2 df (*p* = 0.18) and I^2^ = 42%, indicating a not statistically significant difference in treatment effects across the subgroups analyzed. The moderate I^2^ value suggests some variability between subgroups; however, this variation is not statistically significant and may be attributed to chance.

#### 3.4.4. ∆GT

Three trials reported this outcome (Figure 7) [31,32,72]. I^2^ value was 99%, indicating considerable heterogeneity: the random effect model was thus applied. The difference between groups was not statistically significant (*p* = 0.10; SMD = 2.17; 95% CI from −0.64 to 4.99).

In the subgroups mCAF + sCTG (105 sites) vs. mCAF + XADM (103 sites) and mCAF + sCTG (de-epithelialized) (42 sites) vs. mCAF + sCTG (DBS) (42 sites), the differences were not statistically significant.

The test for subgroup differences yielded a Chi^2^ value of 2.78 with one df (*p* = 0.10) and I^2^ = 64%, indicating that no statistically significant difference was found between subgroups, while the I^2^ value reflects substantial heterogeneity.

#### 3.4.5. ∆KTW

Seven trials reported this outcome (Figure 8) [31,32,33,36,52,70,72]. I^2^ value was 99%, so the heterogeneity was considerable, thus a random effect model was applied. The difference between sCTG and its substitutes was statistically significant, with a *p*-value of 0.005 in favor of mCAF + sCTG.

The test for overall effect of subgroup mCAF + sCTG vs. mCAF + XADM showed a significant difference between groups (*p* = 0.0001) and the I^2^ value was 0%, indicating no observed heterogeneity among the studies.

A statistically significant difference was also observed in mCAF + sCTG vs. mCAF + CM comparison, but the test of heterogeneity was not applicable.

The other comparisons did not show any significant difference between groups.

The test for subgroup differences yielded a Chi^2^ value of 9.68 with 2 df (*p* = 0.008) and I^2^ = 79.3%. This indicates a statistically significant difference between subgroups, with substantial heterogeneity.

#### 3.4.6. RES

Only two trials recorded this outcome (Figure 9) [32,72]: no statistically significant difference was registered between sCTG and adjunctive techniques.

#### 3.4.7. Duration of Surgery

Only two trials recorded the time of surgery (Figure 10) [31,36]: duration of surgery was statistically significant in favor of mCAF adjunctive techniques groups (*p* < 0.00001).

### 3.5. Certainty Assessement

The quality of evidence was assessed for RCTs comparing primary outcomes of mCAF + sCTG vs. mCAF adjunctive techniques (see Table 3).

## 4. Discussion

Coverage of multiple GRs is generally more challenging than treatment of single GRs due to the presence of a larger avascular recipient bed, variable root prominence and shallow vestibule, uneven recession depths, and limited residual keratinized tissue [75].

Compared to the traditional CAF approach, the use of additional grafting, flap technique modifications, or tunnel variation with CTG may improve clinical outcomes [18]. The surgical approach of Modified Coronally Advanced Flap (mCAF) combined with subepithelial connective tissue graft (sCTG) is considered the gold standard; however, assessing and comparing the predictability of alternative adjunctive techniques of mCAF for simultaneous root coverage is challenging due to procedural and material heterogeneity [26]. The use of mCAF + sCTG has shown long-term clinical improvement of recession depth, increasing KT and CAL, besides the morbidity of procedure and the issue concerning graft availability [23].

In this proposal, another meta-analysis already analyzed the additional use of CTG and CM to mCAF: Bathia et al. [26] reported that combining mCAF with either CTG or Collagen Matrix (CM) significantly improved CRC and recession reduction compared to mCAF alone. A more recent systematic review [76] directly compared CAF + CM vs. CAF + CTG and reported a statistically significant difference in favor of sCTG, with superior outcomes in terms of recession reduction (*p* = 0.02) and KTW gain (*p* = 0.03). However, palatal harvesting causes severe discomfort in the palatal area in patients undergoing harvesting [77]; after CTG, patients usually present a small percentage of postoperative complications, such as dentin hypersensitivity, bleeding, edema, pain, and delayed wound healing. Moreover, postoperative pain is a negative experience that causes difficulty in swallowing and negatively influences patient compliance and confidence in treatment [78].

To avoid those complications, it is essential to investigate and evaluate evidence for alternative approaches. Based on recent literature on the association of mCAF with promising biomaterials or other substitutes/approaches, such as EMD [79], this review was conducted to compare the efficacy of different adjunctive techniques, focusing on the treatment of multiple adjacent GR-type defects in terms of root coverage (RC), esthetic outcomes, and keratinized tissue (KT) augmentation. The selection of specific outcomes for a subsequent metanalysis was necessary for a proper comparison, due to the presence of substitutes of different origins, different techniques, and heterogeneous follow-ups.

Only RCTs with a minimum follow-up of 6 months were included, as this duration is considered sufficient for tissues healing after surgery. Studies with longer follow-ups, up to one year, were also considered to account for complete healing [23]. Several biomaterials investigated in this study were superimposable to others analyzed in different systematic reviews [23]: among the included studies, nine evaluating mCAF + sCTG vs. mCAF + other associations (CM, XADM, PRF, EMD, sCTG harvested double blade scalpel) were included in the meta-analysis, with mCAF + sCTG as control group.

Regarding RC, most of the studies (14 out of 17) reported the evaluation of CRC, which was usually considered in other systematic reviews [21,61] as a primary outcome, in terms of both clinical stability and esthetics. CRC was described with early and long-term follow-ups (between 6 months and 5 years) as between 43% and 93% in the case of traditional approaches of mCAF (mCAF alone or with CTG) [27,31,32,33,35,36,38,44,52,68,70,71,72,73,74]; between 50% and 82% in the case of use of substitutes [31,32,33,35,36,38,44,52,71,72,73]. Nevertheless, results for techniques alternative to sCTG are not available with follow-ups longer than 1,5 years: this issue can be underlined as critical for a proper comparison between different approaches, as longer outcomes, even up to 5 years, are declared as stable only with traditional approaches (percentages of CRC around 90%) [74].

Another parameter frequently considered for RC [22] was percentage of MRC, reported in almost all studies: only a few studies [52,70,71], whose approach was the association to mCAF of OB application, XADM, and EMD, revealed percentage of MRC comparable to those of mCAF alone or mCAF + sCTG, but more evidence of these techniques is needed. Similarly to CRC, MRC values of alternative techniques were comparable to traditional approaches of mCAF only in short-term follow-ups, with outcomes up to 95,28% in case of use of sCTG between 6 and 18 months [70]. On the other hand, in long-term follow-ups, it is not possible to compare them with CTG, which remains stable at 91% after 5 years [74]. It can be thus assumed that CRC and MRC well point out the rate of reduction in the initial recession, showing that use of new biomaterials can lead to promising clinical outcomes, despite sCTG still remaining the gold standard in long-term assessment.

Cairo et al. [80] examined esthetic- and patient-related outcomes, finding that CTG procedures showed highest overall esthetic performance for root coverage, although graft integration might impair soft tissue color and appearance. Additionally, CTG-based techniques were also correlated with greater patient satisfaction and morbidity. In this SR, the esthetic performance was evaluated measuring the RES parameter, which provides an objective attempt both for patients and clinicians to categorize esthetic assessment [55], finding a higher RES specifically in the case of using orthodontic buttons [44,71]. However, a high level of evidence showed no statistically significant difference in RES between sCTG and adjunctive techniques.

The ∆GT parameter [31,32,68,72,73], evaluated in only five studies, presented heterogeneous values for all techniques analyzed. Better indicative of a clear tendency, the evaluation of ∆KTW was reported in almost half of studies (10 out of 17) [26,27,28,31,33,58,60,61,62,63], with similar values in terms of CTG (between 0.62 and 1.8 mm) compared to other approaches (between 0.3 and 2.13 mm) considering earlier follow-ups, with no results at longer follow-up.

Regarding comparison between mCAF + sCTG and mCAF + PRF, MRC was found in favor of the control group (*p* = 0.01; SMD = −0.67, 95% CI [0.14, 1.20]). Outcomes regarding PRF still represent a controversial issue, as PRF influences the healing process of both soft and hard tissues, releasing, in time, platelets, growth factors, and cytokines [81]. Despite methods of its production not being standardized, PRF does not require anticoagulants, thrombin, or any other gelling agent, making it easily usable [82]. In this regard, PRF was initially employed in implant surgery to improve bone healing, alveolar ridge preservation, 1- and 2-stage sinus floor elevation, horizontal and vertical bone regeneration, periodontal defects regeneration, third molar extractions, and later used also in plastic periodontal surgery [35,50,69,83,84]. The comparison between the use of PRF membranes added to mCAF versus mCAF alone showed no significant benefits [38] in favor of PRF, except for increasing KT thickness.

Regarding comparison between mCAF + sCTG and mCAF + CM, only one trial reported this comparison [36]: CRC did not demonstrate a statistically significant difference (*p* = 0.05) between groups, while ∆KTW revealed a statistically significant difference (*p* = 0.0006) in favor of the control group (SMD = 0.79, 95% CI [0.34, 1.24]). In the comparison between mCAF + sCTG and mCAF + XADM the parameter CRC was evaluated in four RCTs [31,32,33,70], and a statistically significant difference (*p* = 0.002) was found in favor of the control group (OR = 2.08, 95% CI [1.30, 3.33]). Statistically significant differences again in favor of the control group were found also for MRC (three RCTs [31,32,70], *p* = 0.03) and ∆KTW (four RCTs [31,32,33,70] (*p* = 0.0001). The potential proliferation of fibroblasts may be due to the presence of important growth factors: different collagen matrices were employed to treat gingival recessions associated with dental wear, as autogenous or xenogeneic substitutes, e.g., porcine acellular dermal matrix [31], which consists of 3D sterilized pure collagen types I and III and elastin, a stable tissue matrix derived from porcine dermis without being posteriorly cross-linked artificially or put under any other chemical treatment [85]. In vitro and in vivo investigations showed an increase growth and proliferation of human gingival fibroblasts, osteoblasts, and endothelial cells, revealing a revascularization of the collagen structure during early healing. Furthermore, other authors suggested that it enhances migration, adhesion, and proliferation of periodontal ligament cells and human oral fibroblasts [86,87,88].

Finally, duration of surgery was significant shorter with mCAF adjunctive techniques (*p* < 0.00001): as described by other SR [89], it could represent a determining factor in reducing patient morbidity.

In light of these considerations, to ensure appropriate decision-making, the clinicians should consider patients’ anxiety, discomfort, preference, and esthetics, and discuss with them all the advantages and drawbacks of CTG and its substitutes.

### Limitations of the Study

Considerable variability across the included RCTs is acknowledged, as studies with a split-mouth design introduce a distortion: one intervention’s effect could contaminate the other side of the mouth, making it difficult to estimate the true effect of each treatment. Furthermore, there is an evident heterogeneity of follow-ups (from a minimum of 6 months to 5 years).

Regarding the heterogeneity of interventions included in this SR, only one RCT [74] showed stable long-term results of CTG in terms of RC, plus the association of sCTG to mCAF also reached better results in the long-term for KTW augmentation. On the other side, a lack of evidence in the long-term emerged regarding the alternative approaches of mCAF for RES, MRC, KTW gain, and GT gain. A low level of evidence suggested that mCAF + EMD and mCAF+ sCTG (DBS) may provide good results.

As the meta-analysis revealed these heterogeneous outcomes, study limitations derive from discrepancy in follow-ups between comparisons, not homogeneous distribution of techniques analyzed, and different study design performed, with unclear blinding of participants or examiners in few cases.

Furthermore, regarding studies on the association with PRF, the investigation by Culhaoglu et al. [59] dealt with the PRF technique employed in two different modalities, two or four layers, respectively. In this case, by the union of the two groups, a single mean and standard deviation were considered to avoid potential bias for data duplication.

Future perspectives in the study search could focus on targeted reviews based on comparable techniques in terms of follow-ups of at least 3 years and similar study design.

## 5. Conclusions

Based on the findings of this SR and meta-analysis, the combination of the mCAF with a sCTG remains the most effective and evidence-supported approach for the treatment of multiple adjacent gingival recessions, ensuring superior and stable long-term outcomes for RC, RES, and KT gain.

The mCAF combined with biomaterials or adjunctive techniques (such as XADM, PRF, orthodontic button, or EMD) demonstrated promising short-term results in clinical outcomes; however, the lack of long-term data and the high heterogeneity among studies prevent drawing definitive conclusions on their clinical predictability. Despite this, some mCAF adjunctive techniques can be associated with a significant reduction in duration of surgical procedures, which may contribute to decrease patient morbidity and thus improve procedural efficiency. In terms of esthetic outcomes (RES), alternative mCAF approaches also showed favorable results, as no statistically significant differences were found when compared with the use of sCTG.

Further RCTs with a parallel-groups design and a follow-up of more than 5 years are needed to reliably assess the clinical effectiveness and predictability of these alternatives compared to the established use of sCTG.

## Figures and Tables

**Table 1:** Summary and characteristics of the included studies in the SR.

Authors	Year	Country	Design	N° Patients	N° Sites	Surgery Test vs. Control	Clinical Outcomes	Follow-Up
Alexiou et al. [52]	2017	Greece	RCT (split mouth)	12	54	mCAF + sCTG vs. mCAF + EMD	CRC, MRC	6 months
Aroca et al. [38]	2009	France	RCT (split mouth)	20	67	mCAF + sCTG vs. mCAF	CRC, MRC	6 months
Cairo et al. [68]	2016	Italy	RCT (parallel groups)	32	74	mCAF vs. mCAF + sCTG	CRC, RDR, KTW, GT, duration of surgery, RES	12 months
Culhaoglu et al. [69]	2018	Turkey	RCT (parallel groups)	22	63	mCAF + sCTG vs. mCAF + PRF (2 and 4 layers)	MRC	6 months
George et al. [39]	2018	India	RCT (split mouth)	15	60	mCAF + placental membrane vs. mCAF	RDR, KTW	6 months
Gurlek et al. [33]	2019	Turkey	RCT (split mouth)	12	82	mCAF + XADM vs. mCAF + sCTG	CRC, RDR, KTW	1,5 years
Khobragade et al. [44]	2016	India	RCT (split mouth)	20	116	mCAF + OB application vs. mCAF	CRC, MRC, RES	6 months
Maluta et al. [70]	2021	Brazil	RCT (split mouth)	15	94	mCAF + sCTG vs. mCAF + XDM	CRC, MRC, RDR, KTW	6 months
Meza-Mauricio et al. [32]	2021	Brazil	RCT (parallel groups)	41	130	mCAF + XADM vs. mCAF + sCTG	CRC, MRC, RDR, KTW, GT, RES	12 months
Nahas et al. [36]	2020	Brazil	RCT (split mouth)	15	82	mCAF + CM vs. mCAF + sCTG	CRC, MRC, RDR, KTW, duration of surgery	12 months
Öncü, E. [35]	2017	Turkey	RCT (split mouth)	30	60	mCAF + Platelet-Rich Fibrin (PRF) vs. mCAF + sCTG	CRC, MRC	6 months
Ozcelik et al. [71]	2011	Turkey	RCT (parallel groups)	41	155	mCAF vs. mCAF + OB	CRC, MRC, RDR, KTW, RES	6 months
Özkan Şen, D. and Öncü, E. [50]	2023	Turkey	RCT (parallel groups)	20	118	mCAF + T-PRF (Titanium Platelet-Rich Fibrin) vs. mCAF + sCTG	MRC	6 months
Ragghianti Zangrando et al. [72]	2021	Brazil	RCT (split mouth)	21	84	mCAF + sCTG (harvested de-epithelialized) vs. mCAF + sCTG (harvested double blade scalpel DBS)	CRC, MRC, RDR, KTW, GT, RES	6 months
Rotundo et al. [73]	2019	Italy	RCT (parallel groups)	24	61	mCAF vs. mCAF + CM	CRC, MRC, RDR, KTW, GT, duration of surgery	12 months
Santamaria et al. [31]	2023	Brazil	RCT (parallel groups)	38	78	mCAF + XADM (NCCLs) vs. mCAF + sCTG (NCCLs)	CRC, MRC, RDR, KTW, GT, duration of surgery	6 months
Zucchelli et al. [74]	2014	Italy	RCT (parallel groups)	50	149	mCAF vs. CAF	CRC, duration of surgery	5 years

**Table 2:** Outcomes of individual studies included in the SR.

Study	Surgical Technique	CRC (%)	MRC (%)	RDR (mm)	∆KTW (mm)	∆GT (mm)	RES (0–10)	Duration of Surgery (min)
Alexiou et al. [52]	MCAF + EMD	55	81.7	/	0.58	/	/	/
	MCAF + CTG	63	79.7	/	1.23	/	/	/
Aroca et al. [38]	MCAF	74.62	80.7	/	/	/	/	/
	MCAF + PRF	52.23	91.5	/	/	/	/	/
Cairo et al. [68]	MCAF	47	/	2.4	−0.4	0.002	7.9	54.7
	MCAF + CTG	83	/	3.0	1.8	0.66	7.9	79.4
Culhaoglu et al. [69]	MCAF + PRF (2 and 4 layers)	/	63.00	/	/	/	/	/
	MCAF + CTG	/	80.13	/	/	/	/	/
George et al. [39]	MCAF + Placental membrane	/	/	1.206	2.133	/	/	/
	MCAF	/	/	1.225	0.677	/	/	/
Gurlek et al. [33]	MCAF + XADM	70.7	/	2.40	0.92	/	/	/
	MCAF + CTG	87.8	/	2.50	0.75	/	/	/
Khobragade et al. [44]	MCAF	43.8	78,3	/	/	/	7.57	/
	MCAF + OB	77.9	92.23	/	/	/	8.76	/
Maluta et al. [70]	MCAF + XADM	75.5	92.68	2.43	0.74	/	/	/
	MCAF + CTG	82.6	95.28	2.54	0.91	/	/	/
Meza-Mauricio et al. [32]	MCAF + XADM	70.3	80.19	2.39	0.63	0.54	8.12	/
	MCAF + CTG	83.3	91.79	2.75	0.99	0.77	8.31	/
Nahas et al. [36]	MCAF + CM	60	77.78	2.00	0.3	/	/	31.3
	MCAF + CTG	68	82.14	2.20	1.2	/	/	47.7
Öncü, E. [35]	MCAF + PRF	50	77.12	/	/	/	/	/
	MCAF + CTG	60	84	/	/	/	/	/
Ozcelik et al. [71]	MCAF	61	89.1	3.89	0.66	/	7.43	/
	MCAF + OB	84.6	96.2	4.65	0.48	/	8.65	/
Özkan Şen, D. and Öncü, E. [50]	MCAF + PRF	/	61.77	/	/	/	/	/
	MCAF + CTG	/	75.31	/	/	/	/	/
Ragghianti Zangrando et al. [72]	MCAF + CTG (de-epithelialized)	38	82.5	2.07	0.62	0.55	8.04	/
	MCAF + CTG (double blade scalpel)	38	78	2.09	0.84	0.64	8.36	/
Rotundo et al. [73]	MCAF + CM	63	87	2.0	0.6	0.2	/	36.1
	MCAF	52	75	2.0	1.1	−0.3	/	47.3
Santamaria et al. [31]	MCAF + XADM	50.7	63.6	1.57	0.3	0.30	/	37.4
	MCAF + CTG	72.9	91.3	2.30	0.96	0.90	/	52.7
Zucchelli et al. [74]	MCAF	78.08	/	/	/	/	/	29.8
	MCAF + CTG	90.78	/	/	/	/	/	40.2

**Table 3:** Certainty assessment using GRADEpro GDT. Explanations: ^a^ The difference between the two groups was statistically significant; however, the test for heterogeneity was not calculated as only one study was included in this subgroup. Therefore, the effect of this intervention cannot be considered generalizable. ^b^ the test for heterogeneity was not calculated as only one study was included in this subgroup. Therefore, the effect of this intervention cannot be considered generalizable. ^c^ The standard deviation was not reported. ^d^ There was a considerable heterogeneity of total events.

Outcomes	№ of Participants (Studies) Follow-Up	Certainty of the Evidence (GRADE)	Comments
CRC (%)	604 (7 RCTs)	⨁⨁⨁⨁ High	sCTG shows a stat. sig. higher CRC than adjunctive technique, with a high level of evidence.
CRC (%)—mCAF + sCTG vs. mCAF + XADM	384 (4 RCTs)	⨁⨁⨁⨁ High	sCTG shows a stat. sig. higher CRC than XADM, with a high level of evidence.
CRC (%)—mCAF + sCTG vs. mCAF + CM	82 (1 RCT)	⨁⨁◯◯ Low ^a^	sCTG shows a non-stat. sig. higher CRC than CM, with a low level of evidence.
CRC (%)—mCAF + sCTG (de-ep) vs. mCAF + sCTG (DBS)	84 (1 RCT)	⨁⨁◯◯ Low ^b^	sCTG (de-ep) and sCTG (DBS) showed no difference in CRC, with a low level of evidence.
CRC (%)—mCAF + sCTG vs. mCAF + EMD	54 (1 RCT)	⨁⨁◯◯ Low	sCTG shows a non-stat. sig. lower CRC than EMD, with a low level of evidence.
KTW gain (mm)	557 (7 RCTs)	⨁⨁⨁⨁ High	sCTG shows a stat. sig. higher KTW gain than adjunctive technique, with a high level of evidence.
KTW gain (mm)—mCAF + sCTG vs. mCAF + XADM	337 (4 RCTs)	⨁⨁⨁⨁ High	sCTG shows a stat. sig. higher KTW gain than XADM, with high level of evidence.
KTW gain (mm)—mCAF + sCTG vs. mCAF + CM	82 (1 RCT)	⨁⨁⨁◯ Moderate ^b^	sCTG shows a stat. sig. higher KTW gain than CM, with a moderate level of evidence.
KTW gain (mm)—mCAF + sCTG (de-ep) vs. mCAF + sCTG (DBS)	84 (1 RCT)	⨁⨁⨁◯ Moderate ^b^	sCTG (de-ep) shows a non-stat. sig. slightly lower KTW gain than sCTG (DBS), with a moderate level of evidence.
KTW gain (mm)—mCAF + sCTG vs. mCAF + EMD	54 (1 RCT)	⨁◯◯◯ Very low ^b,c^	The SMD between sCTG and EMD was not estimable because no standard deviation was reported. This comparison, based solely on mean values, provided very low level of evidence.
RES	214 (2 RCTs)	⨁⨁⨁⨁ High	There is no stat. sig. difference between the RES of sCTG and that of adjunctive techniques, with a high level of evidence.
RES—mCAF + sCTG vs. mCAF + XADM	130 (1 RCT)	⨁⨁⨁◯ Moderate ^b^	sCTG shows a non-stat. sig. slightly higher RES than XADM, with a moderate level of evidence.
RES—mCAF + sCTG (de-ep) vs. mCAF + sCTG (DBS)	84 (1 RCT)	⨁⨁⨁◯ Moderate ^b^	sCTG (de-ep) shows a non-stat. sig. slightly lower RES than sCTG (DBS), with a moderate level of evidence.

## Data Availability

Data are available from the corresponding author upon reasonable request.

## Abbreviations

The following abbreviations are used in this manuscript:

ADM	Acellular Dermal Matrix
CAF	Coronally Advanced Flap
CAT	Coronally Advanced Tunnel
CI	Confidence Interval
CM	Collagen Matrix
CRC	Complete Root Coverage
CTG	Connective Tissue Graft
DBS	Double Blade Scalpel
df	Degree of Freedom
EMD	Enamel Matrix Derivatives
GR	Gingival Recession
GRADEpro	GDT Guideline Development Tool
GT	Gingival Thickness
HA	Hyaluronic Acid
I2	Index of Higgings
KT	Keratinized Tissue
KTW	Keratinized Tissue Width
MAGRs	Multiple Adjacent Gingival Recessions
MCAF	Modified Coronally Advanced Flap
MRC	Mean Root Coverage
NCCLs	Non-Carious Cervial Lesions
OB	Orthodontic Button
OR	Odds Ratio
PICOS	Population, Intervention, Comparison, Outcomes, and Study Design
PRF	Platelet-Rich Fibrin
RC	Root Coverage
RCPPS	Root Coverage Periodontal Plastic Surgery
RCSPs	Root Coverage Surgical Procedures
RCTs	Randomized Clinical Trials
RDR	Recession Depth Reduction
RES	Root Coverage Esthetic Score
Robvis tool	Risk of Bias Visualization Tool
RoB2	Risk of Bias 2
RR	Risk Ratio
RT	Recession Type
sCTG	Subepithelial Connective Tissue Graft
SMD	Standardized Mean Difference
SRs	Systematic Reviews
VAS	Visual Analogue Scale
T-PRF	Titanium Platelet-Rich Fibrin
XADM	Xenogeneic Acellular Dermal Matrix
